# The Rice Eukaryotic Translation Initiation Factor 3 Subunit e (OseIF3e) Influences Organ Size and Pollen Maturation

**DOI:** 10.3389/fpls.2016.01399

**Published:** 2016-09-20

**Authors:** Wenyi Wang, Mengyun Xu, Xuejiao Liu, Jumin Tu

**Affiliations:** Institute of Crop Science, College of Agriculture and Biotechnology, Zhejiang UniversityHangzhou, China

**Keywords:** OseIF3e, translation initiation, *OsICKs*, *Oryza sativa* L., reproductive development, pollen maturation

## Abstract

Eukaryotic translation initiation factor 3 (eIF3) is a large protein complex that participates in most translation initiation processes. While eIF3 has been well characterized, less is known about the roles of individual eIF3 subunits, particularly in plants. Here, we identified and characterized *OseIF3e* in rice (*Oryza sativa* L.). *OseIF3e* was constitutively expressed in various tissues, but most strongly in vigorously growing organs. Transgenic *OseIF3e*-silenced rice plants showed inhibited growth in seedling and vegetative stages. Repression of *OseIF3e* led to defects in pollen maturation but did not affect pollen mitosis. In rice, eIF3e interacted with eIF3 subunits b, d, e, f, h, and k, and with eIF6, forming homo- and heterodimers to initiate translation. Furthermore, OseIF3e was shown by yeast two-hybrid assay to specifically bind to inhibitors of cyclin-dependent kinases 1, 5, and 6. This interaction was mediated by the sequence of amino acid residues at positions 118–138, which included a conserved motif (IGPEQIETLYQFAKF). These results suggested although OseIF3e is not a “functional core” subunit of eIF3, it still plays crucial roles in rice growth and development, in combination with other factors. We proposed a pathway by which *OseIF3e* influence organ size and pollen maturation in rice, providing an opportunity to optimize plant architecture for crop breeding.

## Introduction

In the process of translation initiation, eukaryotic initiation factors (eIFs) participate in the recruitment of initiator tRNA (Met-tRNA_i_^Met^) and mRNA to the 40S ribosomal subunit, as well in scanning for the AUG start codon (Browning et al., 2001; Kapp and Lorsch, 2004; Hinnebusch, 2006). Of the 12 known eIFs, eukaryotic translation initiation factor 3 (eIF3) is the largest and most complex. It is involved in assembling the eIF2-GTP-Met-tRNA_i_^Met^ ternary complex and recruiting it to the 40S subunit, recruiting mRNA to the 43S pre-initiation complex, and scanning for and recognizing AUG start codons (Burks et al., 2001; Kawaguchi and Bailey-Serres, 2002; Siridechadilok et al., 2005; Hinnebusch, 2006).

Mammalian eIF3 contains 13 non-identical subunits designated eIF3a–m (Asano et al., 1997; Browning et al., 2001). In contrast, eIF in *Saccharomyces cerevisiae* comprises only six subunits (eIF3a, eIF3b, eIF3c, eIF3g, eIF3i, and eIF3j). Five of these (eIF3a, eIF3b, eIF3c, eIF3g, and eIF3i) are conserved in all eukaryotes (Phan et al., 1998; Browning et al., 2001). The non-conserved nature of subunit e indicates that it may not be essential for translation initiation (Asano et al., 1998; Burks et al., 2001; Zhou et al., 2005; Xia et al., 2010).

The eIF3e subunit, also known as *Int6*, is a common integration site for the mouse mammary tumor virus (MMTV) genome (Marchetti et al., 1995), which plays multiple roles in translation, as indicated by its association with the COP9 signalosome (CSN). The CSN is known to be involved in the regulation of proteolysis (Yahalom et al., 2001), control of 26S proteasome activity (Yen et al., 2003), and spindle organization (Yen and Chang, 2000; Morris and Jalinot, 2005). These findings suggest its potential as a regulatory subunit for gene translation (von Arnim and Chamovitz, 2003).

Few studies have examined the functions of the various eIF3 subunits in plants, some of those have been conducted in *Arabidopsis* (Kim et al., 2004; Yahalom et al., 2008; Xia et al., 2010). Two *Arabidopsis thaliana eIF3e* mutants are known (Yahalom et al., 2008). *AteIF3e-Tp*, which carries an insertion (T) 150 bp upstream of the first exon, leads to reduced fertility and reproductive defects (Yahalom et al., 2008). The mutant *eIF3e-Tnull*, containing an insertion (T) in the middle of the third exon, results in lethality of the male gametophyte. These results suggest that *AteIF3e* is necessary for male gametogenesis. Mutations in subunits *eIF3f* and *eIF3h* have also been characterized in *Arabidopsis*. A *Ds* (transposon element) insertion mutation in *AteIF3f* has been found to disrupt pollen germination and embryonic development (Xia et al., 2010). Plants homozygous for *AteIF3h* mutation exhibit pleiotropic growth defects throughout development, including low fertility, reduced stamen number, partial seed abortion, and inhibition of root hair formation (Kim et al., 2004, 2007). Subsequently, Zhou et al. (2014) described a mutation in *AteIF3h* led to expansion of shoot apical meristem (SAM) size accompanied by a failure to initiate new organs. Recently, the biological function of OseIF3f has been studied by Li et al. (2016). The OseIF3f-RNAi plants showed a higher percentage of arrested unicellular pollen at bicellular stage and aborted pollen at the tricellular stage, it is suggest that OseIF3f plays a vital role in microgametogenesis. Overall, even eIF3 subunits are not part of the functional core, it’s also play important roles in the growth and development of *Arabidopsis* and rice (Li et al., 2016).

Organ size is controlled by two fundamental processes: cell proliferation and cell expansion, which are strictly regulated by cyclin-dependent kinases (CDKs) together with their specific cyclin partners (Mizukami and Fischer, 2000; Sugimoto-Shirasu and Roberts, 2003). Other factors act as inhibitors of CDK (ICK) during plant development and in response to environmental changes (Sherr and Roberts, 1999). Studies in plants, particularly *Arabidopsis* and rice, have shown that overexpression of various *ICK* genes results in phenotypic effects similar to those produced by mutations of eIF3 subunits, including small organ sizes, reduced cell numbers, pollen sterility, and low seed setting (Wang et al., 2000; De Veylder et al., 2001; Zhou et al., 2002; Barroco et al., 2006; Bemis and Torii, 2007; Kang et al., 2007). For example, overexpression of either *AtICK1* or *AtICK2* in *Arabidopsis* induces cells to initiate endoreduplication earlier than normal, resulting in a higher ploidy numbers (Verkest et al., 2005; Weinl et al., 2005). Similarly, overexpression of rice *OsiICK6* results in multiple phenotypic effects on plant growth, pollen viability, and seed setting (Yang et al., 2011).

A previous study revealed that inhibition of *Osj10gBTF3* (*Oryza sativa* BTF3) results in dramatic plant miniaturization. Furthermore, pollen is completely sterile, an effect correlated with the altered expression of two Rf (fertility restorer)-like genes encoding pentatricopeptide repeat-containing proteins (OsPPRs); two translation initiation factors, OseIF3e and OseIF3h; and the heat shock protein OsHSP82 (Wang et al., 2012). The present study sought to confirm the functions of *OseIF3e* in plant growth and development. Specifically, protein–protein interactions demonstrated that OseIF3e plays important roles in rice growth and pollen development and interacts with eIF3 subunits OseIF3b, OseIF3d, OseIF3e, OseIF3f, OseIF3h, OseIF3k, as well as eIF6 and ICKs. Taken together, these results help to unravel a possible pathway for *OseIF3e* involvement in organ growth and pollen development in rice.

## Materials and Methods

### Plant Materials, Growth Conditions, and Stress Treatments

Rice (*O. sativa* L ssp *japonica* cv Nipponbare) was used in this study for various experiments. All plants were grown on the experimental field of Zhejiang University in Hangzhou (30°16′N, temperate climate, China) or Sanya (19°2′N, tropical climate, China) during the natural growing season. Rice seedling plant were grown at 28°C with 16 h light/8 h dark cycle, 75% relative humidity in greenhouse. For expression studies of *OseIF3e* in response to various treatments, 2-week-old seedlings were transferred to Yoshida solution (Yoshida et al., 1976) supplemented with 200 mM NaCl, 10 μM ABA, 100 μM GA. Seedlings grown in the same liquid medium without any supplementary component were used as controls. For cold stress, 4-week-old seed-derived seedlings were transferred from semi-solid 1/2MS medium (Murashige and Skoog, 1962) to Yoshida solution, were exposed in 4°C for 24 h.

### Vector Construction and Rice Transformation

To generate *OseIF3e* knock-down transgenic lines, the *OseIF3e* cDNA fragments of 325 bp (from 174 to 499 bp of *OseIF3e*, Supplementary Figure S3) was inserted into pTCK303 vector (Wang et al., 2004) to produce RNAi repression vectors. The resultant vector was introduced into *Agrobacterium tumefaciens* strain EHA105, which was used to infect rice embryogenic calli from Nipponbare. Transgenic plants were screened by PCR amplification with hygromycin B phosphotransferase gene (*Hpt*). All primers used in this study are listed in Supplementary Table S2.

### Phenotypic Analysis of Transgenic Plants

The evaluation of phenotypic traits of three independent transformants *OseIF3e*^Ri^-2, *OseIF3e*^Ri^-4, *OseIF3e*^Ri^-7 were performed in the T1–T3 generation. Seeds of *OseIF3e*^Ri^ and wild-type (WT) plants were collected and germinated by soaking in water for 2 days at 37°C. Germinating seeds were sown in experimental field as described above during the natural growing season at five-leaf and maturity stage, the phenotypic characteristics were measured and photographed, including plant height, tiller number, the internode length, panicle length, the spikelet number, the grain length and width. The data were analyzed by ANOVA, and mean values were separated by least significant difference at the 5 and 1% probability level using Statistical software (Sigmaplot10.0.).

### RNA Extraction, cDNA Synthesis, and Quantitative Real-Time RT-PCR

Total RNAs were extracted from different tissues of the WT and *OseIF3e*^Ri^ plant using TRIzol reagent (Invitrogen). Reverse transcription (RT) was performed using SuperScript III Reverse Transcriptase (Invitrogen) according to the manufacturer’s instructions. Quantitative real-time RT-PCR (qRT-PCR) analysis was conducted with the Lightcycler 480 machine using SYBR Green I (TAKARA). *UBIQUITIN* (*Os03g0234200*) mRNA was used as an internal control. The specific primers for qRT-PCR are listed in Supplementary Table S2.

### Yeast Two-Hybrid Assay

The yeast two-hybrid assay was performed using the Matchmaker Two-Hybrid System (Clontech^1^). The full-length CDS and different truncations of OseIF3e, OsICKs, and other subunits of OseIF3, OseIF1, OseIF2, OseIF4, OseIF5, and OseIF6 were amplified by PCR using the primers listed in Supplementary Table S2. The fragments were cloned into the pGBKT7 or pGADT7 vector. Then co-transformed into yeast strain AH109 first selected on SD/-Leu/-Trp (DDO) plates at 30°C for 3 days, signal colony from yeast transformants including different pair of constructs were diluted in 0.9% NaCl, and a 1/10th dilution was spotted on SD/-Ade/-His/-Leu/-Trp (QDO) plates and incubate at 30°C for 3 days. Yeast cells co-transformed with pGBKT7-53 and pGADT7-T were used as the positive control, pGBKT7-Lam and pGADT7T were used as the negative control.

### I_2_-KI and DAPI Staining

To analyze pollen viability, mature anthers were incubated with 1% (w/v) I_2_-KI staining, with three biological repetitions. The stained pollen grains were observed and recorded using a Leica DMIRB fluorescence microscope. For 4′,6-diamidino-2-phenylindole (DAPI) staining, pollen grains were fixed in DAPI staining solution (0.1 M sodium phosphate, pH 7.0, 1 mM EDTA, 0.1% Triton X-100 and 0.25 mg/ml DAPI) for 1 h at room temperature. Photography was performed using Leica DMIRB fluorescence microscope under UV light.

### Bioinformatics Analysis

To investigate gene’s structure, the exon/intron boundary were predicted with RGAP^2^, and protein domains were predicted by PROSITE^3^, PLACE^4^ was used for analysis *cis*-elements of *OseIF3e* promoter region. The primers used in this study were designed by primer primer5.0 and the BLAST^5^ was used for sequence alignment. Alignment was performed using CLUSTALX1.8 (Thompson et al., 1994) with default settings. All amino acid sequences were obtained from the NCBI database^6^. Phylogenetic analysis was conducted using MEGA5 via the neighbor-joining method (Kolaczkowski and Thornton, 2004). Motif 1,2 in OsICK1,-5,-6 and consensus sequence of the conserved motif in eIF3e from different species using were identified by the MEME/MAST program^7^ (Bailey and Elkan, 1994; Bailey and Gribskov, 1998).

## Results

### Characteristics and Expression Patterns of *OseIF3e*

**FIGURE 1:**
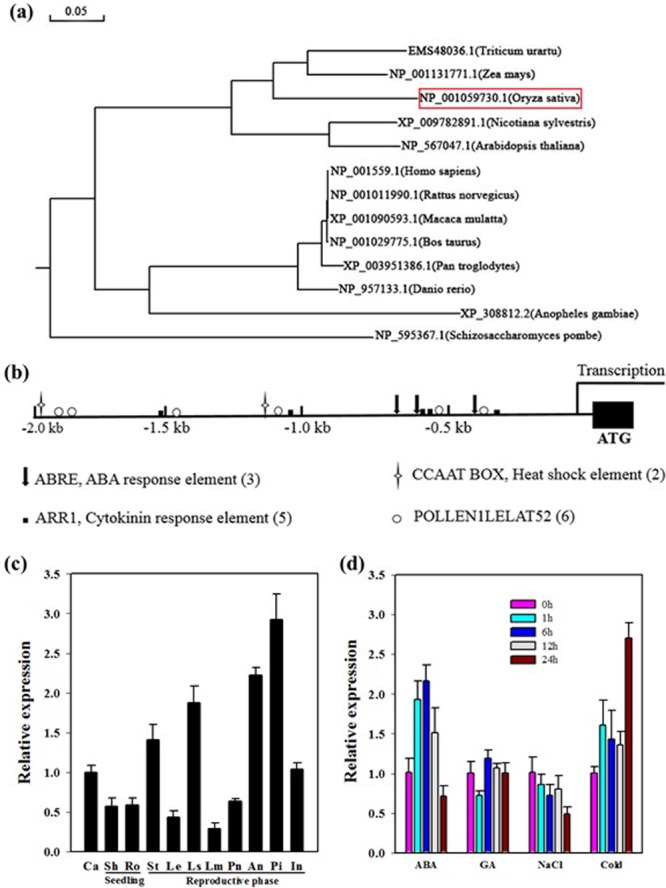
**Characteristics and expression profile of *OseIF3e*. (a)** Phylogenetic tree constructed by MEGA5 software using the neighbor-joining method. **(b)** *Cis*-element analysis of the *OseIF3e* promoter region. **(c)** Expression of *OseIF3e* in various organs. Ca, callus; Sh, shoot; Ro, root; St, stem; Le, leaf; Ls, sheath; Lm, lemma; Pa, palea; An, anther; Pi, pistil; In, internode. **(d)** Expression of *OseIF3e* under hormone and stress treatments, including ABA, GA, salt, and cold (0, 1, 6, 12, 24 h) treatments in seedlings.

In rice, *OseIF3e* was originally identified via its interaction with the basal transcription factor Osj10gBTF3, inhibition of which results in plant miniaturization and pollen abortion (Wang et al., 2012). Previous studies have characterized a multitude of eIF3e homologs from other species. We constructed a phylogenetic tree of *OseIF3e* according to sequence homology. This revealed that OseIF3e is most closely related to ZmeIF3e, while homologs in animals and fungi form separate clades (**Figure 1a**).

To investigate the expression profile of *OseIF3e*, we searched the CREP (Collection of Rice Expression Profiles) database^8^, which collects genome-wide expression data over the life cycles of two rice varieties (Wang et al., 2010). This revealed *OseIF3e* to be constitutively expressed in all tissues and organs, with particularly high expression levels in young and developing tissues (Supplementary Table S1 and Figure S1). We then performed qRT-PCR to confirm *OseIF3e* expression patterns in the following tissues: callus (Ca), shoot (Sh), root (Ro), stem (St), leaf (Le), sheath (Ls), lemma (Lm), palea (Pa), anther (An), pistil (Pi), and internode (In). The results were consistent with the CREP data, with higher *OseIF3e* expression occurring in vigorously growing tissues (**Figure 1c**). These results implicate *OseIF3e* in both vegetative growth and reproductive development in rice.

Next, we analyzed the 2.0-kb promoter region of *OseIF3e* and found several types of *cis*-acting elements, including several hormone response elements. These included three ABREs, five ARR1s (cytokinin response elements), and two heat shock elements (**Figure 1b**). Accordingly, we performed qRT-PCR to determine *OseIF3e* expression levels under different hormonal and abiotic stress treatments in seedlings (**Figure 1d**). The results showed *OseIF3e* to be induced by cold, but repressed by salt treatment. For the ABA treatment, *OseIF3e* transcripts increased within the first 6 h, but then decreased. GA treatment only slightly affected *OseIF3e* expression.

### Transgenic *OseIF3e*-Silenced Rice Plants Show Inhibited Growth in Seedling and Vegetative Stages

**FIGURE 2:**
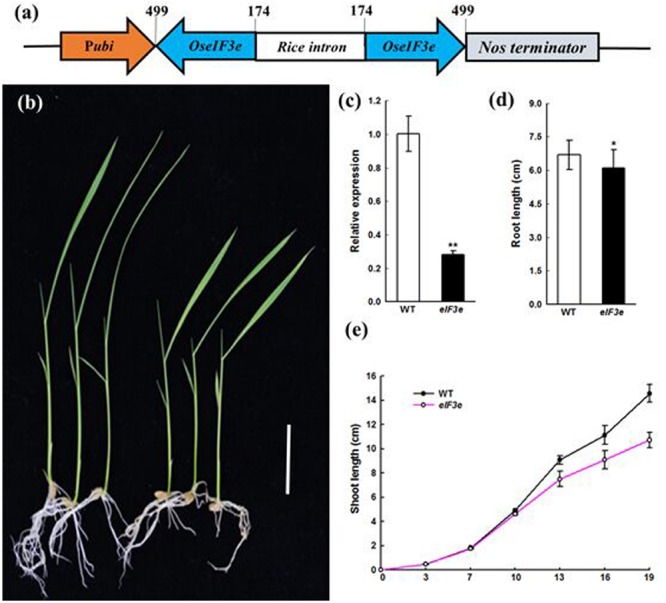
**Phenotype of the *eIF3e* knockdown seedling. (a)** Schematic representation of the *OseIF3e* RNAi vector construction. The numbers indicate the nucleotide position in the *OseIF3e* CDS sequence. P*ubi*, maize ubiquitin promoter. **(b)** Wild-type (WT, left) and *OseIF3e*^Ri^ plants (right) at 15 days after sowing. Scale bars: 3 cm. **(c)** Relative expression level of *OseIF3e* in WT and *OseIF3e*^Ri^ plants. Two-week-old seedlings were used for the analysis. Rice ubiquitin (*OsUBQ*) was used as the reference. **(d)** Two-week-old seedlings root length of WT and *OseIF3e*^Ri^ plants. Values are expressed as means ± SD. ^∗^*P* < 0.05; ^∗∗^*P* < 0.01 compared with the WT plant using Student’s *t*-test. **(e)** Height of WT and *OseIF3e^Ri^* plants after sowing.

To determine the function of *OseIF3e* in rice, we obtained nine *OseIF3e*^Ri^ knockdown lines, in which RNAi reduced the expression of *OseIF3e* (**Figures 2a,c**). Eight transgenic plants had significant decreases in *OseIF3e* compared with WT plants. Then three independent transformants *OseIF3e*^Ri^-2, *OseIF3e*^Ri^-4, *OseIF3e*^Ri^-7 were used for further experiments. Within the first 10 days after germination, these lines did not differ significantly from the WT in terms of seed germination and phenotypic expression (**Figure 2c**). *OseIF3e*^Ri^ lines gradually became slower than that of the WT (**Figure 2e**), leading to shorter shoots (**Figure 2b**) and slightly shorter roots (**Figure 2d**) in OseIF3e plants. When the plants entered the vegetative period, other organs in the *OseIF3e*^Ri^ plants were reduced, e.g., the length and width of the first flag leaf were shorter than in the WT (Supplementary Figure S2). No differences were observed in tiller number. Overall, before maturity, *OseIF3e*^Ri^ transgenic plants differed most markedly from the WT in seedling and flag leaf phenotypes.

These phenotypic differences between WT and *OseIF3e*^Ri^ plants remained stable in generations T_0_–T_3_, confirming that they were indeed due to the suppression of *OseIF3e* (**Figure 2c**). The observation that *OseIF3e* suppression led to stunted rice suggests that *OseIF3e* is critical to the growth of seedling and vegetative-stage plants.

### Aberrant Panicle Phenotype and Low Plant Biomass in *OseIF3e*^Ri^ Lines

**FIGURE 3:**
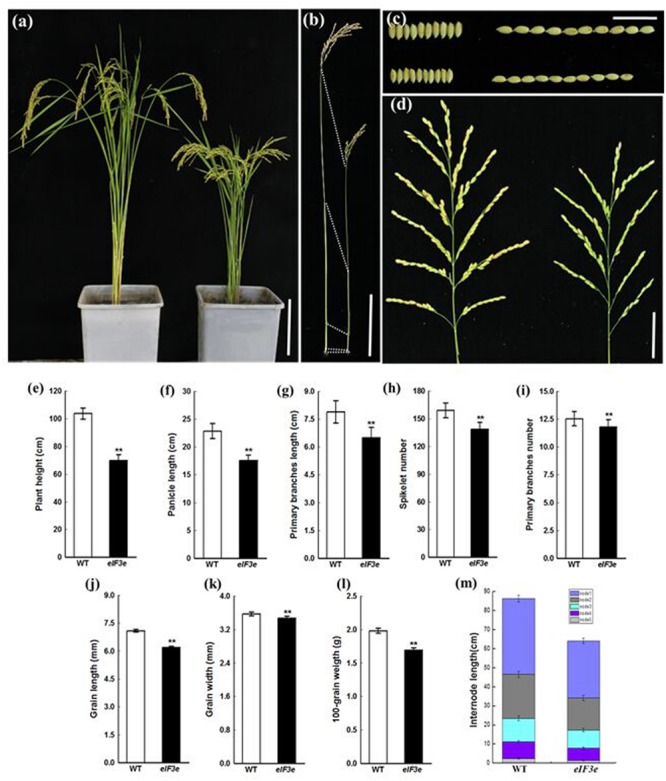
**Phenotype and statistical analysis of panicle and seed of the *OseIF3e*^Ri^ plant at the maturity stage. (a)** Five-month-old wild-type (WT, left) and *OseIF3e*^Ri^ (right) plants. Scale bars: 20 cm. **(b)** Comparison of the internode in WT (left) and *OseIF3e*^Ri^ (right) plants. Scale bars: 4 cm. **(c)** Seed width and seed length in WT (upper) and *OseIF3e*^Ri^ (lower) plants. Scale bars: 2 cm. **(d)** Panicle branching in WT (left) and *OseIF3e*^Ri^ (right) plants. Scale bars: 5 cm. **(e)** Statistical analysis of plant height in WT and *OseIF3e*^Ri^ plants. **(f–i)** Statistical analysis of panicle types in WT and *OseIF3e*^Ri^ plants. **(j–l)** Statistical analysis of seed size in WT and *OseIF3e*^Ri^ plants. **(m)** Comparison of internode length of the main culm in WT and *OseIF3e*^Ri^ plants. Values are expressed as means ± SD. ^∗∗^*P* < 0.01 compared with the WT using Student’s *t*-test.

*OseIF3e*^Ri^ plants remained notably shorter than WT plants due to reduced internode lengths (**Figures 3a,b,m**). At the mature stage, the panicle axis of *OseIF3e*^Ri^ was notably shorter than in WT (**Figure 3d**). We measured the lengths of panicle and primary branches and numbers of primary branches and spikelets. The *OseIF3e*^Ri^ plants displayed shorter panicles and reduced spikelet numbers (**Figures 3e–i**). Moreover, the grains of *OseIF3e*^Ri^ lines appeared thinner and shorter than WT grains, resulting in lower 100-grain weights (**Figures 3c,j–l**). These results demonstrated that *OseIF3e* influences not only panicle size and shape, but also overall plant biomass.

### Repression of *OseIF3e* Affects Pollen Maturation

**FIGURE 4:**
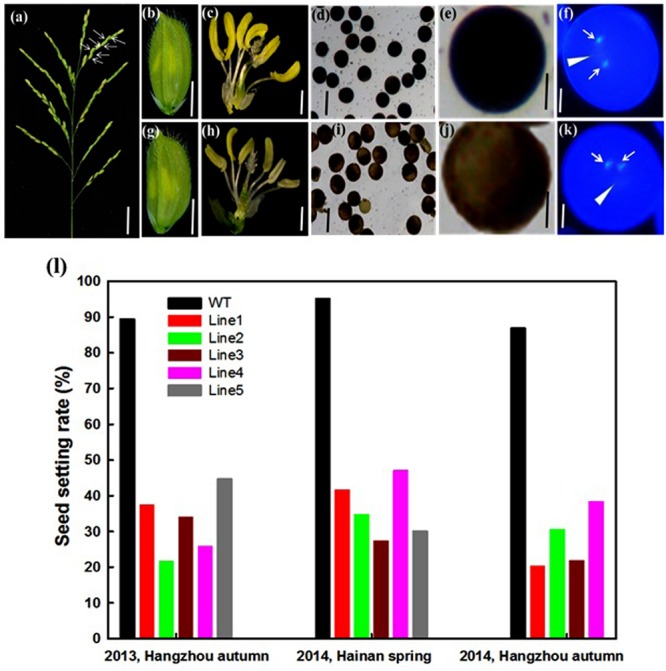
**Analysis of the sterile phenotype in the *OseIF3e* RNAi transgenic plants. (a)** Panicle of *OseIF3e*^Ri^ plants. The white arrow represents empty seeds. Scale bars: 3 cm. **(b,c,g,h)** Wild-type spikelet **(b,c)** and *OseIF3e*^Ri^ spikelet **(g,h)**. The lemma and palea are removed in **(c,h)**. **(d,i)** I_2_-KI staining showing pollen viability in the control plant **(d)** and *OseIF3e*^Ri^ plant **(i)**. **(e,j)** The higher magnification of I_2_-KI staining showing pollen viability in the control plant **(e)** and *OseIF3e*^Ri^ plant **(j)**. **(f,k)** DAPI staining showing three nuclei of pollen grain in the control plant **(f)** and *OseIF3e*^Ri^ plant **(k)**. Arrowheads indicate the vegetative nucleus, and arrows indicate sperm-cell nuclei in the pollen of the control plant **(f)** and abnormal pollen of *OseIF3e*^Ri^ plant **(k)**. **(l)** Seed setting analysis of *OseIF3e*^Ri^ transgenic plants generated in 2013 autumn, and 2014 spring and autumn, respectively. Scale bars: 2 mm in **(b,g)**, 1 mm in **(c,h)**, 100 μm in **(d,i)**, 20 μm in **(e,j)**, and 10 μm in **(f,k)**.

*OseIF3e*^Ri^ plants exhibited a high rate of sterility in generations T_0_–T_3_, which were grown in different locations (**Figures 4a,l**). Seed setting rate of *OseIF3e*^Ri^ plants ranged from 20.2 to 42.8%, compared to from 88.9 to 94.6% in WT plants (**Figure 4l**). In addition, *OseIF3e*^Ri^ plants exhibited abnormal anthers (**Figures 4c,h**). We examined the pollen viability of WT and *OseIF3e*^Ri^ plants with I_2_-KI staining. Stained WT pollen presented full and black, while *OseIF3e*^Ri^ pollen appeared light brown (**Figures 4b,d,e,g,i,j**). To visualize possible mitotic defects, pollen grains were stained with DAPI. DAPI staining revealed two brightly stained sperm nuclei and a large, diffusely stained vegetative cell nucleus in both *OseIF3e*^Ri^ and WT pollen grains (**Figures 4f,k**, arrowhead). Therefore, while repression of *OseIF3e* led to defects in pollen maturation, it did not appear to affect pollen mitosis.

### *OseIF3e*^Ri^ Seedlings Exhibited a Sugar-Sensitive Phenotype

**FIGURE 5:**
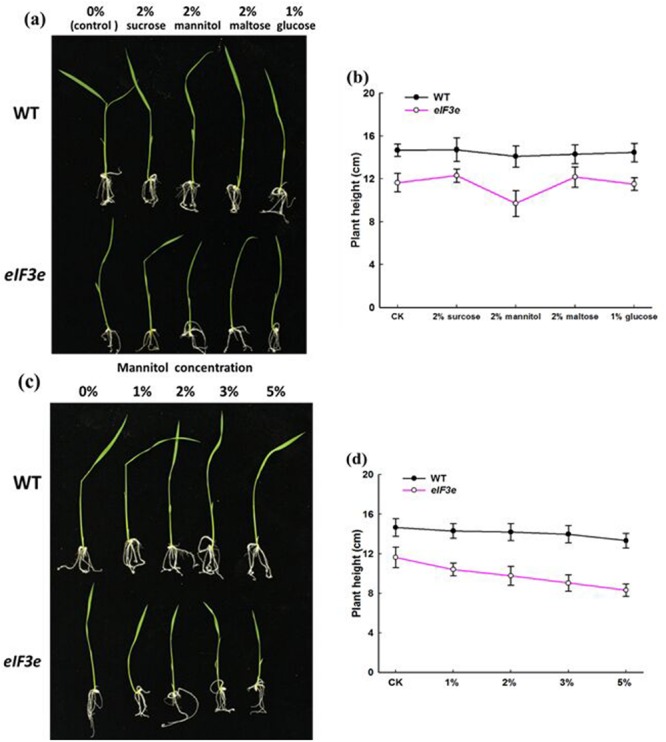
***OseIF3e*^Ri^ seedlings are sensitive to exogenous sugars. (a)** Wild-type (WT) and *OseIF3e*^Ri^ seedlings cultured on 1/2 MS medium containing various sugars. **(b)** Comparison of plant height in WT and *OseIF3e*^Ri^ seedlings cultured on 1/2 MS medium containing various sugars. **(c)** WT and *OseIF3e^Ri^* seedlings cultured on 1/2MS medium containing various concentrations of mannitol. **(d)** Comparison of plant height in WT and *OseIF3e*^Ri^ seedlings cultured on 1/2 MS medium containing various concentrations of mannitol.

In *Arabidopsis*, mutation of either of two eIF3 components, eIF3f and eIF3h, produced a biphasic response to exogenous sugars (Kim et al., 2004; Xia et al., 2010). The present study examined the role of the OseIF3e subunit in response to sugar, using the *OseIF3e*^Ri^ knockdown line. WT and *OseIF3e*^Ri^ seeds were germinated on 1/2MS agar plates containing either no sugar (control) or one of the following: 2% (w/v) sucrose, 2% (w/v) mannitol, 2% (w/v) maltose, 1% (w/v) glucose. The results showed nearly no differences in responses of WT seedlings to sugar treatments. However, *OseIF3e*^Ri^ seedlings exhibited stunted growth in 2% (w/v) mannitol, compared with the other sugar treatments (**Figures 5a,b**). Subsequently, WT and *OseIF3e*^Ri^ seedlings were grown on 1/2MS agar plates containing 0, 1, 2, 3, or 5% mannitol (w/v). As mannitol concentration increased, the growth of *OseIF3e*^Ri^ seedlings appeared more notably stunted, compared to WT (**Figures 5c,d**). In summary, repression of *OseIF3e* caused rice seedlings to become sensitive to exogenous mannitol, resulting in stunted growth of the transgenic plants.

### Yeast Two-Hybrid Assays Reveal that eIF3e Interacts with itself, Other Subunits of eIF3, and eIF6

**FIGURE 6:**
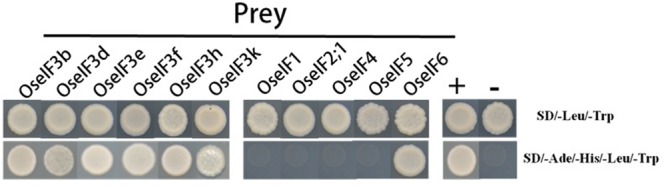
**OseIF3e interacts with itself, other subunits of OseIF3, and OseIF6 protein in Y2H assays.** The bait (BD) vector contained full-length OseIF3e, the prey (AD) vector contained other subunits of OseIF3 and OseIF1,-2,-4,-5,-6, yeast strains were cultured on the QDO (-Ade/-His/-Leu/-Trp) selection medium. p53:T7 (Clontech) and pLam:T7 (Clontech) are positive and negative controls.

The components of eIF3 have been identified in many species. Previous studies show that the different subunits of eIF3 form complexes, which allows them to participate in gene regulation (Kim et al., 2004; Xia et al., 2010). In *Arabidopsis*, eIF3h interacts directly with the eIF3a, eIF3b, eIF3c, and eIF3e subunits (Kim et al., 2004). In addition, the eIF3f subunit has been confirmed to interact with eIF3e and eIF3h (Xia et al., 2010). We performed yeast two-hybrid assays, demonstrating that in rice, eIF3e is able to interact with itself, with other subunits of eIF3 (b, d, f, h, and k), and with eIF6, but does not interact with eIF1, eIF2;1, eIF4, or eIF5 (**Figure 6**). These protein–protein interactions suggest that the subunits of eIF3 and eIF6 form homo- and heterodimers, in different combinations, to initiate translation and regulate target gene expression in rice.

### Targeting of the OsICK Family by the eIF3 Complex is Mediated by Amino Acids 118–138 of eIF3e

**FIGURE 7:**
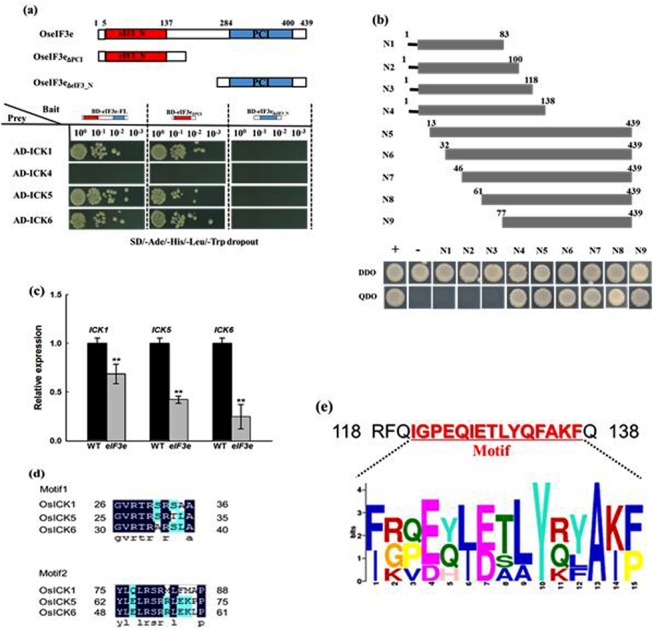
**Interaction of OseIF3e with OsICKs and identification of the binding region responsible for the interaction. (a)** Interaction of OseIF3e with OsICKs in a yeast two-hybrid assay. A schematic diagram of OseIF3e and the truncations. The bait (BD) vector contained full-length OseIF3e, OseIF3e_ΔPCI_, or OseIF3e_ΔeIF3e_N_, the prey (AD) vector contained ICKs (ICK1,-4,-5,-6), yeast strains were cultured on the QDO (-Ade/-His/-Leu/-Trp) selection medium. p53:T7 (Clontech) and pLam:T7 (Clontech) are positive and negative controls. **(b)** The interaction between OsICK5 and a series of truncated version of eIF3e_N domain (N1–N9) in yeast. The bottom panels show the growth of the transformed yeast cells on DDO (-Trp/-Leu) or QDO (-Ade/-His/-Leu/-Trp) selection medium. **(c)** Quantitative RT-PCR analysis of gene expression of *OsICK1, OsICK5*, and *OsICK6.* **(d)** Sequences of conserved motifs in OsICK1, OsICK5, and OsICK6 proteins. **(e)** Consensus sequence of the conserved motif (amino acids 120–137 in rice) in eIF3e from different species. ^∗∗^indicates *P* values generated by student’s *t*-test < 0.01.

To determine whether the OsICK gene family is regulated by the eIF3 complex, the interaction between OseIF3e and OsICKs was investigates by yeast two-hybrid assay. Moreover, considering that the OseIF3e protein possesses relevant domains in its N- and C-terminal regions, we used fragments of OseIF3e encoding the eIF3_N domain (OseIF3e_ΔPCI_), the PCI domain (OseIF3e_ΔeIF3_N_), and the full-length cDNA as baits. The assay revealed that OsICK1, OsICK5, and OsICK6 interacted with OseIF3e and OseIF3e_ΔPCI_, both of which included the eIF3_N domain, while no interaction between OseIF3e_ΔeIF3_N_ and any OsICK was observed (**Figure 7a**). These results suggest that the OsICK family is a direct target of the eIF3 complex and that this interaction is mediated by the eIF3_N domain of OseIF3e.

We then identified conserved sequence motifs in OsICK1, OsICK5, and OsICK6. In these three OsICKs, two consensus sequence motifs were identified by the MEME/MAST program (Bailey and Elkan, 1994; Bailey and Gribskov, 1998; Torres Acosta et al., 2011; **Figure 7d**). Examination of *OsICK1, OsICK5*, and *OsICK6* gene expression in WT and *OseIF3e*^Ri^ lines showed that all three genes experienced various degrees of reduction in *OseIF3e*^Ri^ plants, compared with WT plants (**Figure 7c**).

In order to further characterize the OseIF3e N-terminal motif responsible for its interaction with OsICKs, we cloned fragments encoding different truncations of the OseIF3e N terminus as baits and determined their interaction with OsICK5. As shown in **Figure 7b**, no interaction was detected if the cloned fragment lacked amino acids 118–138 (N4), suggesting that these 20 amino acids which included a conserved motif (IGPEQIETLYQFAKF, **Figure 7e**) are necessary for the interaction to occur.

## Discussion

### *OseIF3e* Is Involved in the Regulation of Organ Size and Pollen Maturation

Plant organ size is controlled by two successive, overlapping types of cell growth: cell proliferation and cell expansion (Mizukami and Fischer, 2000; Busov et al., 2008). To date, several positive and negative factors affecting organ size have been identified in *Arabidopsis* and rice. Positive factors include *AINTEGUMENTA* (*ANT*; Krizek, 1999; Mizukami and Fischer, 2000), ARGOS (Hu et al., 2003), *KLUH/CYP78A5* (Anastasiou et al., 2007), *ORGAN SIZE RELATED1* (Feng et al., 2011), and *XIAO* (Jiang et al., 2012). Negative regulators include *BIG BROTHER* (Disch et al., 2006), PEAPOD1/2 (White, 2006), *DA1* (Li et al., 2008), and *MED25* (Xu and Li, 2011). However, the pathways involved in organ size regulation are not yet well understood.

The present study identified a translation initiation factor in rice, OseIF3e, which we found to influence organ size and pollen maturation. During both the vegetative and reproductive stages, all organs of *OseIF3e*^Ri^ plants exhibited significant reductions in size, compared with WT plants. In addition, repression of *OseIF3e* led to defects in pollen maturation but did not affect pollen mitosis. These results implicate *eIF3e* as an essential gene in rice growth and development.

The *eIF3e* gene was first described as *Int-6*, a common integration site for the MMTV genome (Marchetti et al., 1995). In plants, eIF3e was originally identified as co-purifying with the CSN (Karniol et al., 1998), and its function was verified in *Arabidopsis*. Targeted expression of *AteIF3e* results in pleiotropic effects on development, including defects in seedling, vegetative, and floral development (Yahalom et al., 2008). In this respect, our results are consistent with those reported for *Arabidopsis*. *AteIF3f* and *AteIF3h* mutants also exhibit severe defects in plant growth and development (Kim et al., 2004; Yahalom et al., 2008; Xia et al., 2010). These phenotypes are similar to those of the *OseIF3h*^Ri^ plants examined in our study. Besides, repression of *OseIF3e* led to rice seedlings to become sensitive to mannitol, resulting in stunted growth of *OseIF3e*^Ri^ knockdown lines. These results imply that subunits of eIF3, even though not part of the functional core, are crucial for not only normal plant growth and development, but also abiotic stress response.

### The Activity of the *OseIF3* Complex may be Regulated by OseIF3e in Combination with OseIF3 Subunits b, d, e, f, h, and k, as well as eIF6

Research in plants has shown that different subunits of eIF3 initiate translation and regulate gene expression through the formation of homo- and heterodimers (Karniol et al., 1998; Yahalom et al., 2001; Kim et al., 2004; Huang et al., 2005). For example, in *Arabidopsis*, the eIF3h subunit interacts directly with subunits a, b, c, and e (Kim et al., 2004). Similarly, AteIF3f interacts with both the e and h subunits (Xia et al., 2010).

The present study revealed *in vivo* protein–protein interactions between the OseIF3e subunit and subunits b, d, e, f, h, and k, as well as eIF6. Although it is not part of the highly conserved functional of eIF3, OseIF3e also plays a role in translation processes in combination with other subunits or eIFs. Karniol et al. (1998) first associated subunit eIF3e with the CSN in plants. Further examination by Kim et al. (2004) revealed that AteIF3e interacts directly with AteIF3h. Yahalom et al. (2008) and Xia et al. (2010) subsequently demonstrated that AteIF3e exhibits subcellular co-localization with CSN and is negatively regulated by it. Binding between multiple subunits of eIF3 in *Arabidopsis* suggests the possibility that its activity is regulated by these interactions. However, interactions between eIF3e and other proteins in plants are still largely unknown.

eIF6 was initially identified as a wheat protein capable of interaction with the 60S ribosome (Russell and Spremulli, 1980). In yeast, disruption of *eIF6* results in the abnormal processing of ribosomal RNA precursors and a reduction in abundance of the 60S subunit (Wood et al., 1999; Basu et al., 2001). In *Arabidopsis*, loss of the *AteIF6;1* gene results in embryonic lethality (Kato et al., 2010), suggesting that eIF6 is an essential component of ribosome biogenesis (Si and Maitra, 1999).

### Targeting of *OsICKs* by the *OseIF3* Complex, Mediated by Amino Acids 118–138, Is Responsible for Plant Growth and Development in Rice

We used the proteins OseIF3e, OseIF3e_ΔPCI_ (which included the eIF3_N domain), and OseIF3e_ΔeIF3_N_ (which included the PCI domain) as baits in yeast two-hybrid assays. We thereby determined that three members of the OsICK family (OsICK1, OsICK5, and OsICK6) interacted with OseIF3e and OseIF3e_ΔPCI_, but not OseIF3e_ΔeIF3_N_. This demonstrated that the interactions were mediated by the eIF3_N domain, as the deletion of this region resulted in the lack of interaction. Interestingly, the interaction between eIF3 and CDK was confirmed during apoptosis (Shi et al., 2003), while ICK as inhibitor of CDK which also interact with eIF3, suggesting that eIF3 play a vital role in processes which CDK and ICK participate in, such as cell cycle and cell proliferation.

The ICK family of CDK inhibitors have been identified as key genes in plant growth and development. Seven *ICK* genes, along with a pseudogene, have been reported in rice and several studies have reported notable effects on plant growth and development due to their over-expression (Wang et al., 2000; Barroco et al., 2006; Yang et al., 2011). For example, over-expression of *OsICK6* results in multiple phenotypic effects on plant growth, morphology, pollen viability, and seed setting (Yang et al., 2011). Transgenic overexpression of *OsiICK1* affects endosperm development and greatly reduces seed filling (Barroco et al., 2006). In the present study, we analyzed the phenotypes of *OseIF3e* RNAi-mediated knockdown transgenic rice plants, which revealed pleiotropic growth inhibition throughout development. The phenotypes of the OseIF3e^Ri^ plants were similar to those reported in transgenic plants that overexpress OsICK1 and OsICK6.

Consistent with this finding, in the present study, knockdown of *OseIF3e* dramatically reduced the expression of *OsICK1, OsICK5*, and *OsICK6*, suggesting that *OseIF3e* may influence the cell division cycle via interaction with ICKs. In addition, we found that the OseIF3e amino acids 118–138 are necessary for its interaction with OsICKs. This interaction may be mediated by either motif1 or motif2 in OsICKs and by amino acids residues 118–138 in OseIF3e (**Figure 7d**). The eIF3e subunit may interact with various proteins that possess different binding specificities to initiate translation and regulate the expression genes involved in the development of plants.

**FIGURE 8:**
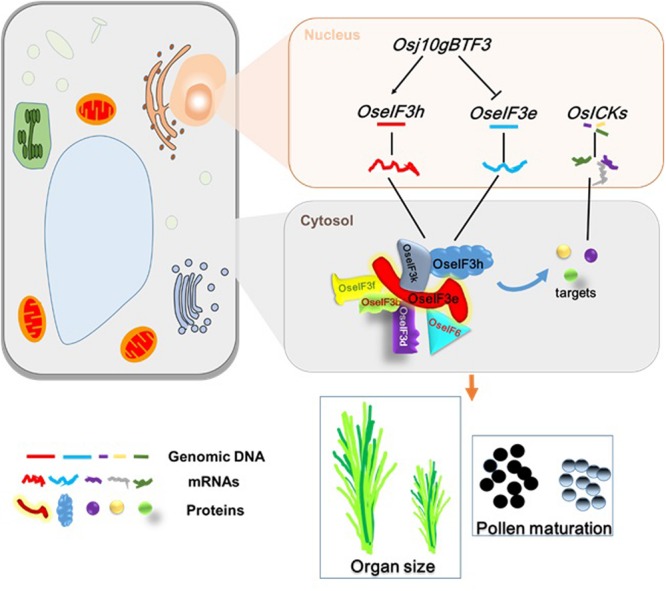
**A general model of *OseIF3e* involved in rice growth and pollen development.** The expression of *OseIF3e* and *OseIF3h* were regulated by *Osj10gBTF3* in the nucleus. In the cytosol, OseIF3e interacts with OseIF3 subunits b, d, e, f, h, k and eIF6 to form homo- and heterodimers. Then, eIF complex binds to OsICKs, thereby affecting their functions and leading to wide-ranging defects.

In summary, this study points to OseIF3e as a crucial regulator of rice seedling development and reproductive processes, including pollen maturation. Based on previous results and the present findings, we propose a possible regulatory model (**Figure 8**) for the role of *OseIF3e* in these processes: (i) *Osj10gBTF3* regulates transcription of *OseIF3e* and *OseIF3h* in the nucleus (Wang et al., 2012); (ii) in the cytosol, OseIF3e interacts with OseIF3 subunits b, d, e, f, h, and k, and with eIF6, to form homo- and heterodimers; (iii) the eIF complex interacts with OsICKs to regulate cell division, affecting plant growth and development. These findings may lead to a better understanding of the factors influencing plant growth and pollen development. Moreover, *OseIF3e*-mediated regulation of *OsICK*s genes pleiotropically modulates several plant characteristics (e.g., plant height, spikelet number, and seed size), providing an opportunity to optimize crop architecture for crop breeding.

## Author Contributions

WW and JT conceived and designed the project, analyzed the data, and wrote the manuscript. MX and XL helped with data analysis. The manuscript was approved by all other authors.

## Conflict of Interest Statement

The authors declare that the research was conducted in the absence of any commercial or financial relationships that could be construed as a potential conflict of interest.

## Footnotes

1 http://www.clontech.com/

2 http://rice.plantbiology.msu.edu

3 http://prosite.expasy.org/

4 http://www.dna.affrc.go.jp/htdocs/PLACE/

5 http://blast.ncbi.nlm.nih.gov/Blast.cgi

6 http://www.ncbi.nlm.nih.gov/

7 http://meme-suite.org/tools/tomtom

8 http://crep.ncpgr.cn

## Abbreviations

ABA: abscisic acid
ABREs: ABA response elements
BTF3: basal transcription factor 3
CDS: coding sequence
eIF3e: eukaryotic translation initiation factor 3 subunit E
eIF3h: eukaryotic translation initiation factor 3 subunit H
GA: gibberellin
ICK: inhibitors of cyclin-dependent kinases
*Os*: *Oryza sativa*
qRT-PCR: quantitative reverse transcription-PCR
RNAi: RNA interference
