# Supplementary material for: The Rice Eukaryotic Translation Initiation Factor 3 Subunit e (OseIF3e) Influences Organ Size and Pollen Maturation
Source: Front Plant Sci. 2016 Sep 20;7:1399. doi: 10.3389/fpls.2016.01399 (PMC5028392; doi:10.3389/fpls.2016.01399)
Supplement: Supplementary file 1 [file Table_1.PDF]

Table S1 Transcript levels of *OseIF3e* at different developmental stages and tissues of variety “Minghui 63” obtained from CREP (http://crep.ncpgr.cn). The signal values represent the average hybridization signal values.

NO.	Developmental stage	Singal value
1	endosperm, 14 days after pollination	1028.8
2	flag leaf, 14 days after heading	1427.3
3	endosperm, 21 days after pollination	2120
4	spikelet, 3 days after pollination	2520.9
5	leaf, 4-5cm young panicle	2650.6
6	sheath, 4-5cm young panicle	2718.5
7	panicle, 4-5cm young panicle	4279
8	plumule, 48h after emergence, dark	3853.8
9	(radicle, 48h after emergence, dark	2469.7
10	plumule, 48h after emergence, light	3166.8
11	radicle, 48h after emergence, light	2932.6
12	flag leaf, 5 days before heading	1595.1
13	stem, 5 days before heading	2374
14	endosperm, 7 days after pollination	1807.2
15	seed, 72h after imbibition	3010.1
16	panicle, heading stage	2338.4
17	stem, heading stage	2374
18	endosperm, 7 days after pollination	1807.2
19	seed, 72h after imbibition	3010.1
20	panicle, heading stage	2338.4
21	stem, heading stage	1553.1
22	hull, one day before flowering	1589.5
23	young panicle at stage 3	3316.5
24	young panicle at stage 4	2678.8
25	young panicle at stage 5	3083.6

26	leaf, young panicle at stage 3	2525.5
27	sheath, young panicle at stage 3	2660.5
28	root, seedling with 2 tillers	2297.2
29	embryo and radicle after germination	1247.5
30	leaf and root, three-leaf stage	1369.1

Table S2 Primers used in this study

Experiments	Primer Name	Sequence 5 ´to 3 ´
Vector constuct	RNAi-eIF3e-F	GATCGAGCTCGGATCC GAACCCTAGAAAACCCTAGCAGC
	RNAi-eIF3e-R	GATCACTAGTGGTACC TTCTGGAGGAAGGAGACGATG
qRT-PCR analysis	qPCR-eIF3e-F	GTTGTTTAGCATTGACTCCGTTAT
	qPCR-eIF3e-R	TTCCGCACTTCACTTCAACAC
	**eIF3e: Os07g0222300**	
	qPCR-eIF3h-F	TTCCGCACTTCACTTCAACAC
	qPCR-eIF3h-R	ATCATAACAGTTGCCACTCATCAC
	**eIF3h:Os04g0376500**	
	qPCR-ICK1-F	GCAACAGCCTCAGCACAGCA
	qPCR-ICK1-R	CACCGGAGTCCACTCGAACC
	**ICK1: Os02g0762400**	
	qPCR-ICK5-F	AGTTCCCGGCGCAGAATG
	qPCR-ICK5-R	AAAGCCTGATGTTGCTGTTGT
	**ICK5: Os03g0137800**	
	qPCR-ICK6-F	GCCACAACATTATTCCAGCATC
	qPCR-ICK6-R	AAGAGGGCAGTCATTCACAGG
	**ICK6: Os10g0471700**	
	UBI-F	CTGTCAACTGCCGCAAGAAG
	UBI-R	GGCGAGTGACGCTCTAGTTC
Y2H	BK-eIF3e-F	GGAATTCCATATGATGGCGGAGCACGACCTGAC
	BK-eIF3e-R	ACGCGTCGACTCACCGAGCTGCCTGTTGTG
	BK-N-eIF3e-F	GGAATTCCATATGATGGCGGAGCACGACCTGAC
	BK-N-eIF3e-R	ACGCGTCGACCAAGTACCTATCCTGGAAGA
	BK-C-eIF3e-F	GGAATTCCATATGTATCAATACCGTGCTTTGTG
	BK-C-eIF3e-R	ACGCGTCGACAGCTTTCGATAACCTGCT
	AD-eIF3b-N-F	GGAATTCCATATGTTCGGCATCCTCAGTG

AD- eIF3b-N-R	CCGCTCGAGAGCAAATCGCATTAGACG
**eIF3b:Os10g0569200**	
AD-eIF3d-F	GGAATTCCATATGCAGAACAACCGCTCCCACC
AD-eIF3d-R	CGGAATTCTGCCTCCAGTCAACACCAGTAA
**eIF3d:Os05g0566500**	
AD-eIF3e-F	GGAATTCCATATGATGGCGGAGCACGACCTGAC
AD-eIF3e-R	CCGCTCGAGTCACCGAGCTGCCTGTTGTG
AD-eIF3f-F	GGAATTCCATATGAACATCTGCGACAGCTACG
AD-eIF3f-R	CCGCTCGAGCTTCTCGGCTATGCTAATC
**eIF3f:Os05g0104800**	
AD-eIF3h-F	GGAATTCCATATGATGGCGAATCCGGCAGC
AD-eIF3h-R	CCGCTCGAGCTAGTCCTCCTGCAAGGC
AD-eIF3k-F	GGAATTCCATATGATGGCGAGCGAGCAGGCG
AD-eIF3k-R	CGGAATTCCAGCTAAGGACAGGGAAT
**eIF3k:Os03g0182700**	
AD-eIF1-Nde-F	GGAATTCCATATG ATGTCTGATCTCGACATTC
AD-eIF1-Xho-R	CCGCTCGAG TGTTCCTTCTTCACAATG
**eIF1:Os07g0529800**	
AD-eIF2-Nde-F	GGAATTCCATATG TCTGGCCGAGGACTGAT
AD-eIF2-Xho-R	CCGCTCGAG AGGGCAACCTTCTCACCTTT
**eIF2:Os12g0176800**	
AD-eIF4-Nde-F	GGAATTCCATATG GAGAAGCCCACTCTTGGA
AD-eIF4-Xho-R	CCGCTCGAG AGATTGCCTGCCCTTTGG
**eIF4:Os11g0414000**	
AD-eIF5-Nde-F	GGAATTCCATATG GCTCTGCAAAACATTGGTG
AD-eIF5-EcoR1-R	CGGAATTC ACTCTTCCTCGGATTCAG
**eIF5:Os09g0131400**	
AD-eIF6-Nde-F	GGAATTCCATATGATGGCGACCCGTATTCAGTT
AD-eIF6--Xho-R-R	CCGCTCGAGTTACACATAACTGTCGATCA
（N1）BK-eIF3e-1-250-Sal-R	ACGCGTCGAC CCCTGAGCCTGGAGACGAC
（N2）BK-eIF3e-1-299-Sal-R	ACGCGTCGAC TGCTGGTTCTGGAGGAAGG
（N3）BK-eIF3e-1-352-Sal-R	ACGCGTCGAC CCTGGAGCATGTGGAGGTT
（N4）BK-eIF3e-1-414-Sal-R	ACGCGTCGAC AACTGAAACTTGGCAAACTGAT
（N5）BK-eIF3e-40-1237-Nde –F	GGAATTCCATATGATGGACTGCCACCTGGTGT
（N6）BK-eIF3e-94-1237-Nde -F	GGAATTCCATATGTACGCGAACAAGGAGAT
（N7）BK-eIF3e-139-1237-Nde -F	GGAATTCCATATGGGGACCAACATGGTGGACTA

（N8）BK-eIF3e-184-1237-Nde –F	GGAATTC<u>CATATG</u>CACGACACCGACGAGGTCC
（N9）BK-eIF3e-232-1237-Nde –F	GGAATT<u>CCATATG</u>GTCGTCTCCAGGCTCAGGG
AD-ICK1-Nde-F	GGAATTC<u>CATATG</u>ATGGGCAAGTACATGAGGAAGT
AD-ICK1-Xho-R	CCG<u>CTCGAG</u>TCAGCTTCGGCTGCTGACC
AD-ICK4-Nde-F	GGAATTC<u>CATATG</u>ATGGCTGCCGCGGCCGCCACCGT
AD-ICK4-EcoR1-R	CG<u>GAATTC</u>TTACAGGCCCACTGCCGTGCCG
AD-ICK5-Nde-F	GGAATTC<u>CATATG</u>ATGGGGAAGTACATGCGGA
AD-ICK5-EcoR1-R	CG<u>GAATTC</u>CTAGCAGTCTAGCCTTGTCCAT
AD-ICK6-Nde-F	GGAATTC<u>CATATG</u>ATGGGCAAGTACATGCGCAAG
AD-ICK6-EcoR1-R	CG<u>GAATTC</u>TCAGTCTAGCTTGACCCATT
