# Supplementary material for: The Rice Eukaryotic Translation Initiation Factor 3 Subunit e (OseIF3e) Influences Organ Size and Pollen Maturation
Source: Front Plant Sci. 2016 Sep 20;7:1399. doi: 10.3389/fpls.2016.01399 (PMC5028392; doi:10.3389/fpls.2016.01399)
Supplement: Supplementary file 2 [file Data_Sheet_1.PDF]

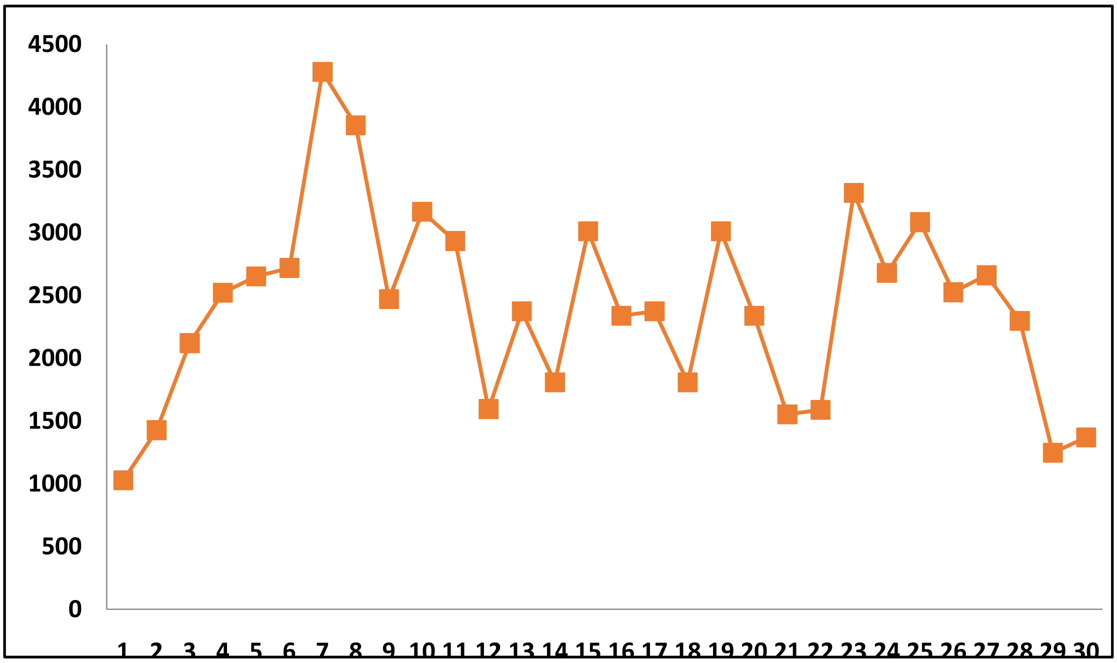

Figure S1 Expression profile of *OseIF3e* gene chip database CREP. The X axis represent the developmental stages indicated in TableS1.The Y axis represent the hybridization signal values.

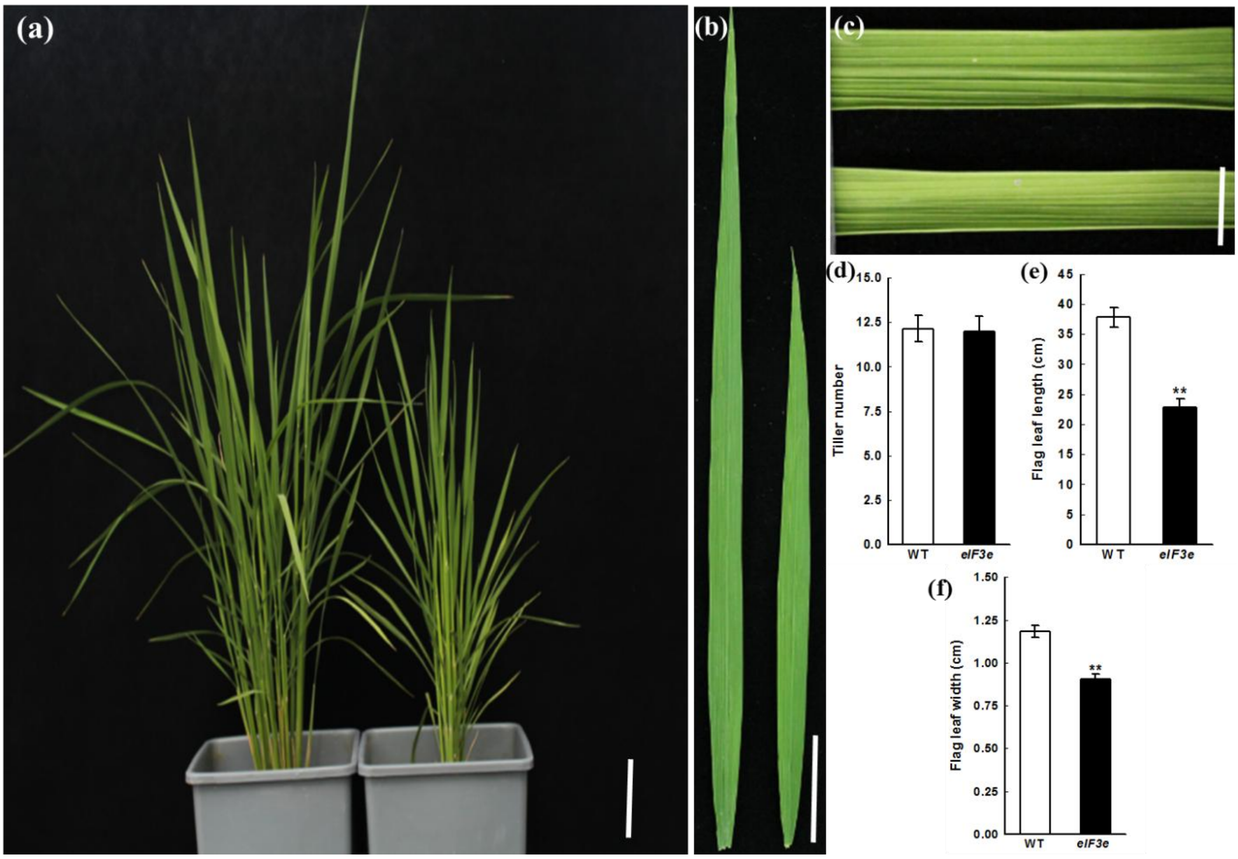

Figure S2. Phenotype of the *OseIF3e*^{Ri}plants at vegetative phase.

(a)Wild-type(right) and *OseIF3e*^{Ri}(left) at 100 days after sowing. Sacle bars:10cm.

(b)Matured leaves in wild-type (left) and*OseIF3e*^{Ri}(right) plants. Sacle bars:5cm.

(c)The width of Matured leaves in wild-type (upper) and *OseIF3e*^{Ri}(lower) plants. Sacle bars:1cm.

(d) Comparison of tillers number per plant in wild-type and *OseIF3e*^{Ri}.

(e, f) flag leaf length(e) and width(f) in wild-type and *OseIF3e*^{Ri}. Values are given as the mean ±SD. **P*<0.05;***P*<0.01 compared with the Wild-type by Student’s *t*-test.

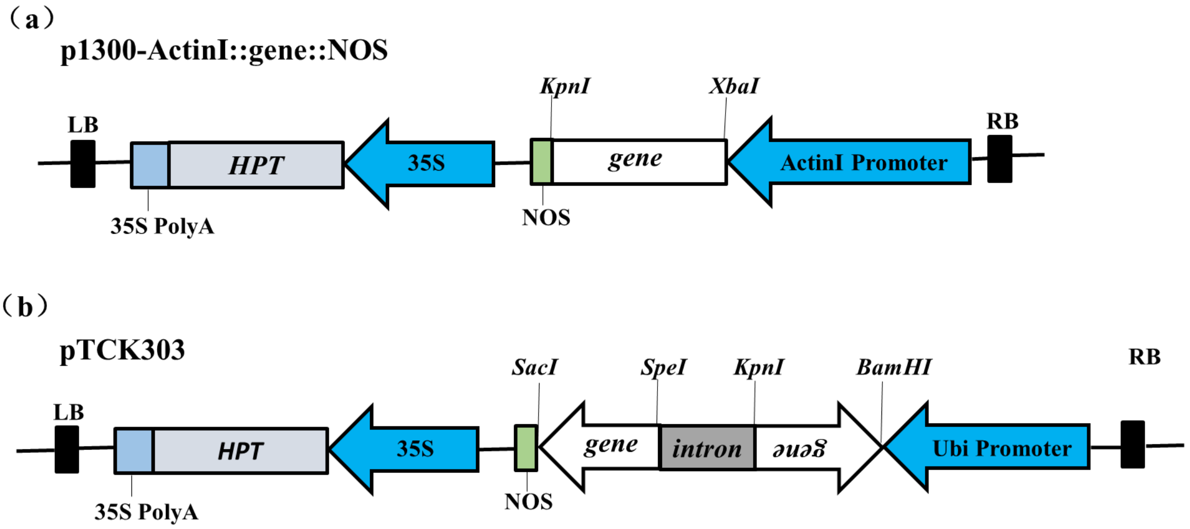

Figure S3 Plasmid constructs used in this study.

(a) For production of overexpression vector, the fragment that wasdigested with *Xba*I and *Kpn*I and cloned into 1300-actin::NOS.

(b) To construct the RNAi vector, the PCR products digested with 2 pairs of restriction enzymes: *Kpn*I/*BamH*I and *SpeI*/*SacI*, respectively. The differently digested fragments were then inserted into pTCK303 (Wang *et al*., 2004).The primers are listed in Supplementary Table S2.
